# Scientists’ warning to humanity for long-term planetary thinking on biodiversity and humankind preservation, a cosmic perspective

**DOI:** 10.1093/biosci/biad108

**Published:** 2024-01-09

**Authors:** Francisco Garcia-Gonzalez, William J Ripple, Aurelio F Malo

**Affiliations:** Department of Ecology and Evolution at Doñana Biological Station (Spanish Research Council), Seville, Spain; Centre for Evolutionary Biology, School of Biological Sciences at The University of Western Australia, Perth, Western Australia, Australia; Department of Forest Ecosystems and Society at Oregon State University and with the Conservation Biology Institute, Corvallis, Oregon, United States; Global Change Ecology and Evolution Research Group, Departamento de Ciencias de la Vida at the Universidad de Alcalá, Alcalá de Henares, Spain; Department of Life Sciences at the Imperial College London, Silwood Park, Ascot, Berkshire, United Kingdom

“We are all made of stars,” the song claims. Indeed, as Carl Sagan said, we are made of star stuff. But we often forget the message of unity ingrained in such reality. The typical narrow short-term perspective characterizing human planning, combined with self-interest, frequently leads to a tragedy of the commons where the very same resources essential for survival or prosperity are compromised or destroyed (Hardin 1968). Such selfish exploitation of common goods sits at the base of many current global problems, including the depletion of natural resources, accelerated climate change, and the loss of biodiversity. Fortunately, there is an increasing awareness of the importance of the natural environment, and an emerging paradigm shift is attempting to ameliorate the negative consequences of global problems for our children and subsequent generations. However, most if not all current plans are somehow shortsighted. For example, the Sustainable Development Goals adopted by the United Nations in 2015 set their targets for the year 2030. The United Nations Climate Change Conferences of the Parties implicitly or explicitly set their purposes for one or a few generations. The question is whether short-term goals and vision will be enough—or even adequate—to ensure the preservation of most life forms, including humanity, into the distant future. Although short-term targets are essential to trigger the necessary immediate action, shouldn't biodiversity preservation thinking and planning occur on a much longer time frame? If so, what is the appropriate time frame?

## What does the future mean?

**Figure 1.**
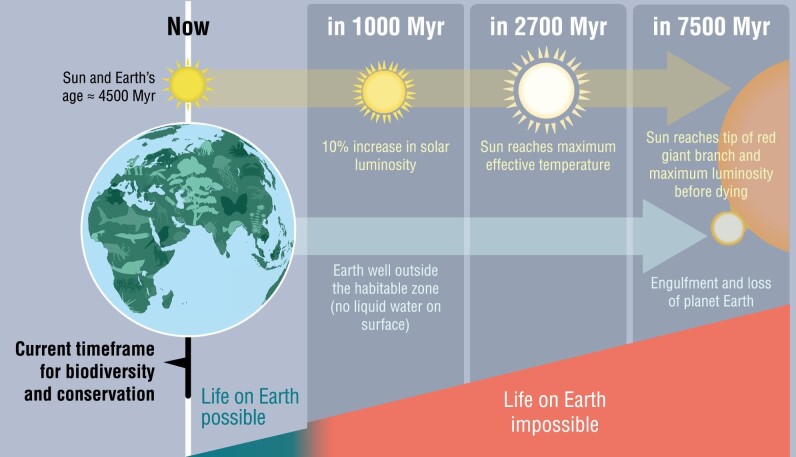
Overview of current short-term and potential long-term (planetary and cosmic) perspectives on conservation. Predictions regarding landmark solar events and the moment Earth will be out of the habitable zone (based on whether the conditions on the planet allow the presence of liquid water on the surface), according to stellar models (Schröder and Connon Smith 2008) and superimposed on a graphical depiction of potential time frames for life on Earth and beyond. A graphical illustration of current time frames for biodiversity and humankind preservation is shown. Time points (in millions of years, Myr) and time frames are given for illustrative purposes and are out of scale.

The current and next few generations are going to be critical to ensure that future generations inherit this planet in a healthy state. If nations manage to come together soon on global matters and we take the necessary actions to prevent the loss of biodiversity on Earth and to ameliorate other worldwide pressing issues (e.g., see table 1), the planet could, in principle, support life, roughly as we know it today, for thousands or millions of years. However, the Sun itself has an end. Astronomers estimate that, in about 5 billion years, the Sun will become a red giant that will absorb Earth (Schröder and Connon Smith 2008). But long before that, because of increased solar activity, within about a billion years, it is expected that life will not be possible on Earth (e.g., because of the evaporation of the oceans; Schröder and Connon Smith 2008). One billion years is about one-fourth of the time that life has been present on Earth; in other words, life on Earth is on its final fifth part of existence. This may sound dismal or fatalistic, but we instead see it as a reality that should spur a deeper sense of unity for humanity toward common goals; this reality reveals the need for thinking not only about global but also transglobal problems, as well as the need for collective and long-term commitments, to allow biodiversity and humanity to go on for as long as possible. The moment at which the conditions on our planet will make life impossible is far away, but we are heading toward it inevitably. When should we start paying attention, collectively, to such reality—1000 years before it happens, 10,000 years before? And when would it be too late? Shouldn't we start thinking about joining forces to allow humanity and as much biodiversity as possible to persist for as long as possible and to even, if feasible, persist beyond the existence of our local solar system? We advocate for the adoption of a cosmic perspective to conservation, including our own conservation (figure 1).

**Table 1.** Relevant climate-related changes and responses, pressing environmental issues, and Earth systems processes.

Variable	Trend
Ozone depletors^a^	**=**
Freshwater resources per capita^a^	**–**
Reconstructed marine catch^a^	**+**
Dead zones^a^	**+**
Total forest area^a^	**–**
Vertebrate species abundance^a^	**–**
Carbon dioxide emissions^a,b^	**+**
Temperature change^a^	**+**
Human population^a,b^	**+**
Ruminant livestock^a,b^	**+**
Total fertility rate^b^	**–**
Per capita meat production^b^	**+**
World gross domestic product^b^	**+**
Global tree cover loss^b^	**+**
Brazilian Amazon forest^b^	**–**
Energy consumption—oil^b^	**+**
Energy consumption—coal^b^	**+**
Energy consumption—gas^b^	**+**
Energy consumption—solar and wind^b^	**+**
Air transport (number of passengers per year)^b^	**+**
Total institutional assets divested^b^	**+**
Per capita carbon dioxide emissions^b^	**+**
GHG emissions covered by carbon pricing^b^	**+**
Carbon price^b^	**+**
Fossil fuel subsidies^b^	**+**
Governments that have declared a climate emergency^b^	**+**
Carbon dioxide^c^	**+**
Methane^c^	**+**
Nitrous oxide^c^	**+**
Surface temperature anomaly^c^	**+**
Minimum Arctic sea ice^c^	**–**
Greenland ice mass change^c^	**–**
Antarctica ice mass change^c^	**–**
Glacier thickness change^c^	**–**
Earth's energy imbalance^c^	**+**
Ocean heat content change^c^	**+**
Ocean acidity^c^	**–**
Sea level change relative to 20-year mean^c^	**+**
Area burned in the United States^c^	**+**
Global tree cover loss due to fires^c^	**+**
Billion-dollar floods in the United States^c^	**+**
Extremely hot days relative to 1961–1990^c^	**+**
Climate change^d^	**>**
Biosphere integrity change^d^	**>**
Stratospheric ozone depletion^d^	*
Ocean acidification^d^	*
Biogeochemical flows (phosphorus and nitrogen cycles)^d^	**>**
Land system change^d^	**>**
Freshwater change^d^	**>**
Atmospheric aerosol loading^d^	*
Novel entities^d^	**>**

*Note:*  ^a^Environmental issues identified in the 1992 scientists’ warning to humanity and for which Ripple and colleagues (2017) analyzed trends from 1960 to the 2010 decade.  ^b^Change in climate-related human activities from 1979 to present times, or ^c^climate-related responses, as in Ripple and colleagues (2023).  ^d^Earth system processes for which planetary boundaries have been established and transgression levels examined (Richardson et al. 2023). The effects and consequences are broader than those shown. In addition to increased global temperatures, droughts, forest fires, extreme weather events, deforestation, altered biogeochemical cycles, local and global extinctions, and rising sea levels, for instance, there is increased soil erosion, loss of biodiversity, imbalances in the phenology and life cycles of organisms, disruption of biological interactions, changes in animal migration patterns, defaunation, increased biological invasions, loss of ecosystem services, in addition to social consequences such as increased chronic and emerging diseases, food crises and famine, displacement of human populations, increased migration, increased poverty and social inequalities, tensions over natural resources, threats to knowledge systems (including indigenous knowledge systems), economic losses, etc. Trends: +, increasing trend; –, decreasing trend; =, approximately unchanged trend; >, planetary boundary transgressed; *, planetary boundary within safe operating space. Simplified trends, from initial to end points, for the time period measured, are given.

The sooner a solution is sought, the better. We are currently in the middle of environmental and climate emergencies (Ripple et al. 2017, Ripple et al. 2023), but we may still be in time to prepare before entering a planetary emergency. An emergency is typically defined by two terms: the importance of the danger and the speed at which the danger arrives. However, a third critical factor is frequently neglected: the speed of the response that allows escaping or coping with the adverse situation. When it comes to the issue at hand, a distant point of no return for the maintenance of biodiversity or any form of life on Earth, the threat is clearly significant. In fact, this is probably the most important danger that humanity will face in its entire existence. However, the danger is quite far away, possibly quite a few millions of years away. But the development of a response capability (sustaining biodiversity up to that time and beyond) appears to be an extremely slow and complex process, because it requires many steps at collective levels, many global agreements and commitments, many technological breakthroughs, and a potentially very long time to test solutions. That is imperative to act now, to protect and bring biodiversity forward the next few generations, is beyond doubt, but it is also crucial to place our gaze on the longer time horizon.

## A change for the better is needed, and it is possible through cooperation

In 1992, a group of more than 1700 independent scientists signed a manifesto warning that humanity was pushing the capacity of ecosystems and Earth to sustain life within acceptable parameters to the limit (Ripple et al. 2017). A second warning from 15,364 scientists 25 years later underscored the failure of nations to establish progress in solving most of the environmental and social problems previously identified and outlined aspects in which the situation worsened quite worryingly (table 1; Ripple et al. 2017). Scientists have provided ample evidence for these issues and other changes brought about or accelerated by human actions with pervasive negative effects on the climate, the ecosystems, and biodiversity, which, in turn, cascade to serious social consequences (table 1; Cardinale et al. 2012, Hoekstra and Wiedmann 2014, Díaz et al. 2019). Moreover, the dangers of surpassing several thresholds that delimit basic conditions for life (planetary boundaries) have also been established, and several of these boundaries have already been transgressed (table 1; Rockström et al. 2009, Richardson et al. 2023). Unfortunately, the list of environmental issues is long and growing. But we may be in time to solve many of the problems (Tallis et al. 2018, Díaz et al. 2019).

Humanity has previously demonstrated that cooperative decisions and actions can make a change for the better on a global scale. Examples include the expansion of renewable energy technologies, the commercial whaling moratorium, and the relative stabilization of the ozone layer as a result of policies regarding the use of substances that destroy it. Another example of progress by human cooperation that can be a landmark for future breakthroughs is the recent exceptional advancement in the development of nuclear fusion (Tollefson and Gibney 2022). The realization that net energy gain may be a reality through nuclear fusion offers hope for high-yield and relatively clean energy in the medium-term future. Concomitantly, obtaining clean, inexpensive, and abundant energy, as is promised by fusion reaction technology, could represent a quantum leap in space exploration potential. Such work on the shoulders of giants illustrates that humans can achieve great accomplishments when the right motivation and investments are in place. Regarding the issue at hand, the questions that require answers are these: Will humans have the motivation, forward-thinking, long-term investment and transnational cooperative attitude required to find solutions to an end-point problem? Or will we be forced to confront a need when it is too late?

## Does it matter?

Many would wonder why we should care about and invest money in distant problems, when a long list of challenging problems predates us incessantly. This has been a recurrent question through human history. If investment is only allocated to pressing, current difficulties, humanity would be in a very primitive, archaic, and undeveloped state. Neil deGrasse Tyson puts it very well with an analogy: “To gain insight, let's rewind thirty thousand years and eavesdrop on our ancestral cave dwellers. Those among them with the urge to explore decide to consult the elders, saying, ‘We want to see what's beyond the cave door.’ The elders are wise. They caucus among themselves, weighing what they think are the risks and rewards, and reply, ‘No. We must first solve the problems of the cave before anyone ventures beyond’” (DeGrasse Tyson 2022). With an exclusive focus on solving imminent problems, which are and will be always around, we would remain unaware that, outside the cave, there may be solutions to solve the problems within the cave.

Some have considered the end of the human lineage, mammals, and most life reflecting geologic and cosmic time scales that are beyond comprehension. If one accepts that we are but a small planet on the edge of a galaxy, with unimaginably more galaxies and planets (around a couple of trillion galaxies following the latest estimations, with each galaxy containing several billion stars and with many stars hosting planets), what does life's extinguishment on Earth really matter? We think that it does indeed matter, if only from a perspective of keeping *maximal cosmic biodiversity*. We also have a moral obligation to try to remedy future problems that may threaten the existence of humanity and life itself. And there are additional nonutilitarian and utilitarian reasons unforeseen today. Regarding nonutilitarian motivations, the possibility of intelligent life throughout the universe is a subject of active debate among astrobiologists (e.g., see Mahecha 2016, Frank 2018), but thus far, there is no scientific evidence of other life in the known universe. Allowing for the only current evidence of life in an inert universe to vanish is therefore unacceptable, if only for ethical reasons. Our duty as a civilization is to give empirical testament of the process of evolution of life on Earth. Even in the event of the discovery of new forms of life, we should shy away from inaction and lame attitudes and do our bit as a civilization to ensure disseminating the empirical example of the story of life on Earth. Second, from a utilitarian standpoint, the preservation of biodiversity on Earth and beyond is a necessity to give our species and the rest of existing biological diversity a life-supporting system. This utilitarian view may also have nonutilitarian consequences, because other extant or future emerging civilizations might be helped by the understanding of our own biological or cultural evolution processes. Under both nonutilitarian and utilitarian views, a plan to maintain biodiversity indefinitely would be required.

## Long-term planetary thinking

Our current generation is witnessing a shift in the collective consciousness of the value of biodiversity and nature. But we must go further. We must have a more distant view of conservation objectives (figure 1). It is perhaps time to think that, at some point, we will need an intergovernmental panel on the long-term future of biodiversity and humanity. We need a planetary plan. The idea of a continuous unity for humankind throughout time has been conceptualized and advocated previously (box 1). However, long-term planetary conceptions for maximal (including humanity) biodiversity conservation are largely lacking (box 1).

Box 1. **Unity of humankind through time.**

Arguably the most prominent defender of unity of humankind through time was Carl Sagan. The description of our planet as Spaceship Earth by Buckminster Fuller is also relevant when considering a planetary conception of humanity. In his view, humanity constituted a group of passengers aboard Spaceship Earth, analogous to a ship's crew who must cooperate in order to keep the ship working. More recently, Neil DeGrasse Tyson and Adam Frank provided enlightening cosmic perspectives on civilization (Frank 2018, DeGrasse Tyson 2022), and the architect Benjamin Bratton advocates in *The Terraforming* for a planetary conception focused on rethinking urban design without forgetting ecosystem preservation (Bratton 2019) and suggests that a Copernican turn is needed in the context of inhabiting Earth. However, long-term planetary conceptions for maximal biodiversity conservation are mostly lacking. The half-Earth suggestion of E. O. Wilson to set aside in reserve at least half the world (Wilson 2016) is arguably the closest initiative attuned with a long-term vision for biodiversity conservation at a global scale. More recent initiatives such as the Global Commons Alliance (https://globalcommonsalliance.org), the Earth Commission (https://earthcommission.org), and Future Earth (https://futureearth.org) have built on this need for global conception, and both the Global Deal for Nature and the Global Safety Net have been proposed as a plan on the basis of scientific evidence for the preservation of life on Earth (Dinerstein et al. 2019) and as an accompanying global-scale analysis of key areas for biodiversity and climate resilience that can serve to inform land use planning (Dinerstein et al. 2020), respectively. We completely agree with Dinerstein and colleagues (2020) that “The level of planning and foresight that is needed to properly scale nature conservation requires the emergence of a worldview that embraces the notion of stewardship at a planetary scale.” All these plans appropriately set or support time-bound milestones (e.g., to conserve at least 30% of the Earth's surface by 2030 and 50% by 2050 or earlier; Baillie and Zhang 2018). We also agree with short- or intermediate-term targets for these actions, because they are mostly needed in the face of the current planetary situation, but we emphasize that they are not in conflict with the longer-term vision for conservation biology that we advocate in this article.

## Conclusions

If we continue with the business-as-usual model, we will be facing, sooner or later, the biggest tragedy of the commons in human history. We can reverse this trend. Humanity needs a sense of unity transcending shortsightedness. If there is a common goal to preserve life and humanity on planet Earth, there is a tangible fundamental reason for us to start working together. Furthermore, we should aim for the common goal of preserving biodiversity even beyond Earth's life, which is finite, inexorably, because of the physics of the solar system (figure 1). Ironically, we need a Copernican turn in the way we consider long-term biodiversity conservation, to take us away from the heliocentrism to which Copernicus and Galileo so properly led us to. We need a starting point to set more ambitious objectives regarding the future of biodiversity. Objectives at short time scales (years, decades, a few generations) are typically set up by governments and decision-makers, and these objectives are needed for tangible actions, but longer-term objectives are also needed. Ensuring that known and not yet described by science biodiversity carries on for as long as possible can be the most important common goal humans would ever face.

## Author Biography

Francisco Garcia-Gonzalez (paco.garcia@ebd.csic.es) is affiliated with the Department of Ecology and Evolution at Doñana Biological Station (Spanish Research Council), in Seville, Spain, and with the Centre for Evolutionary Biology, in the School of Biological Sciences at The University of Western Australia, in Perth, Western Australia, Australia. William J. Ripple is affiliated with the Department of Forest Ecosystems and Society at Oregon State University and with the Conservation Biology Institute, in Corvallis, Oregon, in the United States. Aurelio F. Malo is affiliated with the Global Change Ecology and Evolution Research Group, in the Departamento de Ciencias de la Vida at the Universidad de Alcalá, in Alcalá de Henares, Spain, and with the Department of Life Sciences at the Imperial College London, in Silwood Park, Ascot, Berkshire, United Kingdom.
